# Cardiac RNase Z edited via CRISPR-Cas9 drives heart hypertrophy in *Drosophila*

**DOI:** 10.1371/journal.pone.0286214

**Published:** 2023-05-25

**Authors:** Ekaterina Migunova, Saathvika Rajamani, Stefania Bonanni, Fei Wang, Chao Zhou, Edward B. Dubrovsky

**Affiliations:** 1 Department of Biological Sciences, Fordham University, Bronx, NY, United States of America; 2 Department of Biomedical Engineering, Washington University in St. Louis, St. Louis, MO, United States of America; 3 Center for Cancer, Genetic Diseases, and Gene Regulation, Fordham University, Bronx, NY, United States of America; Biomedical Sciences Research Center Alexander Fleming, GREECE

## Abstract

Cardiomyopathy (CM) is a group of diseases distinguished by morphological and functional abnormalities in the myocardium. It is etiologically heterogeneous and may develop via cell autonomous and/or non-autonomous mechanisms. One of the most severe forms of CM has been linked to the deficiency of the ubiquitously expressed RNase Z endoribonuclease. RNase Z cleaves off the 3’-trailer of both nuclear and mitochondrial primary tRNA (pre-tRNA) transcripts. Cells mutant for RNase Z accumulate unprocessed pre-tRNA molecules. Patients carrying RNase Z variants with reduced enzymatic activity display a plethora of symptoms including muscular hypotonia, microcephaly and severe heart hypertrophy; still, they die primarily due to acute heart decompensation. Determining whether the underlying mechanism of heart malfunction is cell autonomous or not will provide an opportunity to develop novel strategies of more efficient treatments for these patients. In this study, we used CRISPR-TRiM technology to create *Drosophila* models that carry cardiomyopathy-linked alleles of *RNase Z* only in the cardiomyocytes. We found that this modification is sufficient for flies to develop heart hypertrophy and systolic dysfunction. These observations support the idea that the *RNase Z* linked CM is driven by cell autonomous mechanisms.

## Introduction

Cardiomyopathy (CM) is a group of heart diseases that affect cardiac muscle, leading to changes in heart morphology and contractility. It results in various adverse conditions including arrhythmias, heart failure and sudden cardiac death. Heart diseases are the leading cause of death for men and women of all racial and ethnic groups [1]. About 17.9 million people die from a heart disease every year, making up 32% of all deaths worldwide. There are several types of CM, the most prevalent one being the hypertrophic cardiomyopathy (HCM), which is characterized by the thickening of left ventricular wall without lumen dilation. Another common type of CM is dilated cardiomyopathy (DCM), which is described as a dilation with systolic dysfunction of either left or both ventricles [2].

CM affects people of all ages. Adult form of CM is often secondary to inflammation, malnutrition, and other environmental and health conditions [3, 4], while infantile or congenital form occurs due to genetic factors. Infantile CM is etiologically heterogeneous; at least 80 genes have been linked to this group of diseases [5].

Recently the *ELAC2* gene was added to the list of those associated with cardiomyopathy. *ELAC2* encodes a highly conserved RNase Z endoribonuclease which cleaves off the 3’-trailer of both nuclear and mitochondrial pre-tRNA molecules and thereby rendering itself indispensable for tRNA maturation [6, 7]. Mutations in the *ELAC2* gene lead to an especially aggressive form of HCM with early onset of symptoms and lethality within one year of birth [8–11]. In addition to changes in cardiac morphology and function, infants carrying *ELAC2* sequence variants exhibit multiple symptoms including psychomotor and growth retardation, muscular hypotonia, microcephaly and lactic acidosis. Even though the range of clinical manifestations associated with *ELAC2* variants is established, the cellular processes leading up to the heart pathology are not understood. As a result, treatments for *ELAC2* linked CM are still limited to symptom management.

Given the multitude of medical conditions produced by mutations in *ELAC2*, we suggest that this gene is highly pleiotropic affecting multiple organs and organ systems. This phenotypic complexity presents a challenge for finding the origin of key processes that lead to the heart pathology. In many cardiomyopathies, cardiac cells display a cell autonomous response to certain genetic alterations, such as mutations in sarcomere genes that encode proteins forming the major functional unit of myocardium [12–14]. These mutations affect cardiomyocyte contractility, thus causing a direct damage to systolic and diastolic properties of the heart. On the other hand, there are cases of CM that are initiated through non-cell-autonomous mechanisms. For example, non-contractile cardiac fibroblasts produce and release a pro-hypertrophic isoform of the Fgf-2 growth factor, which acts as a paracrine signal on cardiomyocytes to induce overgrowth of a mammalian heart in certain types of HCM [15–17].

For some cases of cardiomyopathy that are associated with inheritable metabolic disorders, the primary cause of cardiac complication has not been definitively established [18]. Propionic acidemia (PA) is one of those inborn disorders caused by the dysfunction of the ubiquitously expressed propionyl-CoA carboxylase (PCC) enzyme [19]. On one hand, the deficiency of PCC causes accumulation of toxic compounds in cardiomyocytes suggesting cell autonomous mechanism of cardiomyopathy. On the other hand, in several cases, liver transplantation led to a complete normalization of cardiac dysfunction in patients with PA [20, 21], which implies that cardiomyopathy associated with PCC deficiency, may develop via non-autonomous mechanism as well.

Like PCC, the RNase Z enzyme is expressed ubiquitously. And while patients with reduced RNase Z enzymatic activity display multiple symptoms, the primary cause of death is acute cardiac decompensation. Knowing whether the underlying mechanism of heart hypertrophy and malfunction is cell autonomous or not might benefit finding the appropriate treatment. It appears that solely analyzing the medical cases of patients carrying *ELAC2/RNaseZ* variants might not provide us with the answer. Thus, we decided to turn to the fruit fly, *Drosophila melanogaster*, a highly tractable genetic model system.

Previously we described a *Drosophila* model of *ELAC2/RNaseZ* linked CM and demonstrated that it exhibits all major symptoms of the human disease [22]. Here, our goal was to establish a specific connection between cell autonomous presence of mutant RNase Z variants in *Drosophila* heart and pathological traits of cardiomyopathy. Using CRISPR/Cas9 technology we generated mosaic flies that carry null and/or missense *RNase Z* alleles in cardiac tube only. Importantly, we found that presence of these alleles in cardiomyocytes is sufficient to cause heart hypertrophy and malfunction. Our findings support the idea that the *ELAC2/RNaseZ* linked CM is driven by cell autonomous mechanisms.

## Results

### Knocking out RNase Z with CRISPR-TRiM

To inactivate cardiac RNase Z we used the CRISPR-mediated tissue-restricted mutagenesis (CRISPR-TRiM) method that was designed to knock out (KO) target genes in any group of cells [23]. This system is based on two transgenes, one that encodes tissue specific Cas9 and another that encodes ubiquitous gRNAs targeting the gene of interest.

**Fig 1:**
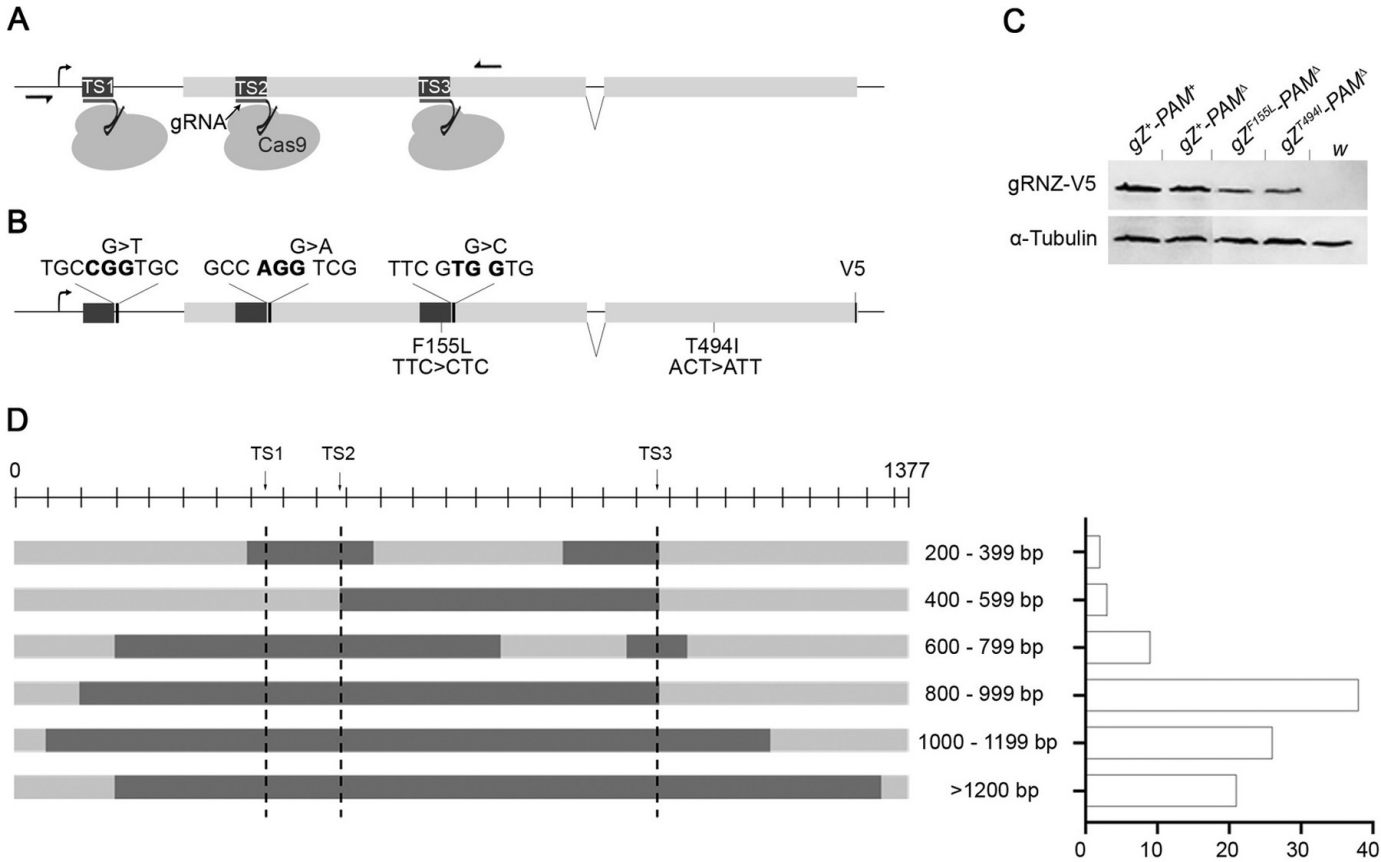
CRISPR-Cas9 mediated knockout of *RNase Z* and Cas9 resistant rescue constructs. (**A**) Schematic illustration of the *RNase Z* locus. Positions of the three target sites (TS1-3) along with the corresponding Cas9:gRNA complexes targeting *RNase Z* are shown (not to scale) relative to the transcription start site (TSS) marked by the standing arrow and the open reading frame marked with the gray horizontal bars. Black horizontal half arrows indicate primers used for the molecular analysis of genomic DNA modifications. (**B**) The V5-tagged RNase Z expression construct carrying single-nucleotide replacements. On the top, nucleotides shown in bold indicate PAM sites; three nucleotide replacements that spoil the PAM sites and make *RNase Z* resistant to Cas9 are also indicated: G>T, G>A, and G>C. At the bottom, the sites of CM-linked mutations (F155L and T494I) are shown with the corresponding nucleotide changes introduced. (**C**) Western blot analysis of RNase Z proteins whose expression is driven by indicated transgenes. The *white*^*1118*^ stock flies are used as a negative control; α-Tubulin is a loading control. RNase Z is detected with the anti-V5 antibody. (**D**) Sequence analysis of modifications introduced by three gRNAs targeting *RNase Z*. The horizontal line illustrates the 1377-bp genomic DNA fragment containing the entire target region along with the ∼350-bp upstream and downstream sequences. A population of these fragments was PCR-amplified on gDNA subjected to CRISPR-Cas9 modification. 100 randomly selected fragments were sequenced and broken into categories based on the size of the deletion they carry– 200/399, 400/599, etc. Representative fragments for every size range are shown with horizontal gray bars, where dark gray indicates deleted nucleotides. Vertical dotted lines mark DSBs at respective target sites drawn to scale. The graph to the right shows the number of fragments in each category.

When Cas9-mediated DNA double-strand breaks (DSB) are repaired via nonhomologous end joining (NHEJ), small insertions and deletions (indels) at or around the target sites are created. Each repaired DSB could result in a loss-of-function mutation. Given that multiple gRNAs increase the chance of gene disruption, we designed three gRNAs all targeting the 5’ end of *RNase Z*, where the indels have the maximum functional impact [24, 25]. Moreover, the presence of multiple DSBs within the same locus could result in a loss of larger DNA fragments in between corresponding cut sites after repair. The frequency of larger deletions increases when gRNAs are located within a few hundred base pairs from each other [23]. To ensure an efficient KO, we designed three gRNAs targeting a 600-bp fragment of the *RNase Z* locus (Fig 1A) and cloned them in a single construct under the ubiquitous U6 promoter. A new line carrying this multi gRNA^Z^ transgene (*U6-3xgRNA*^*Z*^) was established and tested in a cross with flies homozygous for the *Act5C-Cas9* transgene. 100% of F_1_ offspring displayed early lethality, which confirmed the efficiency of multiplexed 3xgRNA^Z^ to knock out expression of the target gene (the same result was achieved using the *tub-Cas9* transgenic line). Importantly, Cas9 mediated *RNase Z* KO larvae (*Act-Cas9*>*RNZ*^*KO*^ and/or *tub-Cas9*>*RNZ*^*KO*^) died during second instar, the stage that was previously described as the effective lethal phase of the *RNase Z* null mutant (*Z*^*24*^) [26]. The consistency in the lethal phases of *Act-Cas9*>*RNZ*^*KO*^ and *Z*^*24*^ highlights the efficacy of the chosen method and suggests that it is indeed the *RNase Z* gene which is removed by CRISPR-TRiM.

One of the well-discussed risks of employing CRISPR technology is the off-target effect which is a nonspecific and unintended genome modification [27–30]. While the three gRNAs were identified and selected as uniquely specific for the *RNase Z* locus, there is still a possibility that Cas9-mediated *RNZ*^*KO*^ larvae could carry modifications elsewhere in the genome that affect their viability. To confirm the specificity of our gRNAs, we turned to a rescue experiment. If early lethality of *Act-Cas9*>*RNZ*^*KO*^ larvae is indeed due to *RNase Z* knock out, this phenotype could be rescued by expression of a wild-type *RNase Z* variant that is resistant to CRISPR/Cas9 cleavage. To produce a DSB, the Cas9 nuclease requires a 3bp DNA sequence (NGG) called the protospacer adjacent motif (PAM) which is present immediately downstream of the target sequence. As per our design, we have two PAM sites–AGG and TGG–present in the open reading frame (ORF), and the third one, CGG, in the 5’UTR of *RNase Z*. To disrupt Cas9 mediated cleavage, we introduced single-nucleotide replacements–G>T, G>A, and G>C–into the PAM sites; the last two are silent mutations within ORF (Fig 1B). A new transgenic line carrying Cas9 resistant *RNase Z* under its natural promoter was generated and designated *gZ*^*+*^*-PAM*^*Δ*^. Western blot analysis confirmed that these three single-nucleotide replacements did not affect RNase Z expression (Fig 1C). Importantly, Table 1, shows that this expression driven by the *gZ*^*+*^*-PAM*^*Δ*^ transgene efficiently rescues *Z*^*24*^ null mutant flies to adulthood, indicating that PAM^Δ^ modifications do not affect RNase Z enzymatic activity and that this protein variant is suitable for the rescue experiment.

**Table 1:** Rescue of Z^24^ null mutants by gZ^+^-PAM^Δ^ transgene.

Parental cross	Progeny genotype ^a^^,^ ^b^^,^ ^c^			
Z^24^/CyO;+/+ x Z^24^/CyO;gZ^+^-PAM^Δ^/TM3,Sb	Z^24^;gZ^+^-PAM^Δ^/+ (n) ^a^^,^ ^b^	Z^24^/CyO;gZ^+^-PAM^Δ^/+ (n) ^b^	Z^24^;TM3,Sb/+ (n) ^a^	Z^24^/CyO;TM3,Sb/+ (n)
	187	417	0	190

^a^ Z^24^ homozygotes were identified by the straight wings (Cy^+^)

^b^ Flies carrying the gZ^+^-PAM^Δ^ rescue transgene were identified by the wild type bristles (Sb^+^)

^c^ The efficiency of rescue was assessed by comparing the number of the rescued progeny (Cy^+^) to their heterozygous siblings (Cy) among offspring that carry the rescue construct (Sb^+^): the expected ratio is 1:2, observed– 187:417

Next, we tested if *gZ*^*+*^*-PAM*^*Δ*^ could rescue the lethal phenotype of *Act-Cas9*>*RNZ*^*KO*^. First, by simple fly crossing we combined two transgenes in one genotype– *U6-3xgRNA*^*Z*^;*gZ*^*+*^*-PAM*^*Δ*^. Next, these flies were crossed to the *Act5C-Cas9* line to produce *Act-Cas9*>*gZ*^*+*^ offspring. As a control, we used *gZ*^*+*^*-PAM*^*+*^ flies that express the unmodified RNase Z protein. Table 2 summarizes results of two crosses. Among the adult offspring of the control cross, we did not observe any females that carry all three transgenes *Act5C-Cas9*, *U6-3xgRNA*^*Z*^ and *gZ*^*+*^*-PAM*^*+*^. At the same time, the expected ratio (according to the Mendelian distribution) of males (that carry *U6-3xgRNA*^*Z*^ and *gZ*^*+*^*-PAM*^*Δ*^ transgenes) to females (that carry *Act5C-Cas9*, *U6-3xgRNA*^*Z*^ and *gZ*^*+*^*-PAM*^*Δ*^ transgenes) was observed in the experimental cross. Thus, the *gZ*^*+*^*-PAM*^*Δ*^ transgene effectively rescues the lethal phenotype of *Act-Cas9*>*RNZ*^*KO*^. This experiment confirms that the only vital gene being affected in *Act-Cas9*>*RNZ*^*KO*^ flies is *RNase Z*. (The same result was achieved using the *tub-Cas9* transgenic line).

**Table 2:** PAM^Δ^ modification makes the *RNase Z* transgene (gZ^+^-PAM^Δ^) resistant to Cas9.

Parental crosses ^a^	Progeny ^b^^,^ ^c^	
	Females (n) ^b^	Males (n)
♀ w; U6-3xgRNA^Z^; gZ^+^-PAM^+^ x ♂ Act-Cas9/Y	0	535
♀ w; U6-3xgRNA^Z^; gZ^+^-PAM^Δ^ x ♂ Act-Cas9/Y	247	268

^a^ The first cross is control, with unmodified *RNase Z* transgene (gZ^+^-PAM^+^); the second cross is experimental that assesses the resistance of the modified *RNase Z* transgene (gZ^+^-PAM^Δ^)

^b^ Only female progeny carries both U6-3xgRNA^Z^ and Act-Cas9 transgenes, and thus, displays the CRISPR-Cas9 mediated knockout of endogenous *RNase Z*

^c^ The resistance of the modified *RNase Z* transgene (gZ^+^-PAM^Δ^) to Cas9 was evaluated by comparing the number of female and male progeny: the expected ratio is 1:1, observed– 247:268

In general, the outcome of the CRISPR-Cas9 mediated gene modification is not uniform in all cells. Each DSB produced by Cas9 is tackled by the DNA repair system leading to a spectrum of modifications; it is particularly true when Cas9 targets a specific locus with multiple gRNAs. To assess the variability in the outcome for our CRISPR-TRiM design, we investigated the specifics of DNA damage introduced in the *RNase Z* locus. Fragments of *tub-Cas9>RNZ*^*KO*^ genomic DNA encompassing the targeted region were PCR-amplified and cloned. 100 such clones were randomly selected and sequenced. Strikingly, this analysis revealed that every cloned fragment carried a deficiency either continuous or discontinuous. Most of the fragments (97/100) displayed a continuous pattern with the size of deletions ranging from 445 bp to over 1200 bp removing the entire sequence between the first and the third target sites (Fig 1D). Less frequently observed was the discontinuous pattern (3/100) wherein the total deletion ranged from 245 bp to 720 bp. Importantly, at least 2 of the target sites were modified in all fragments under the survey, with 78% of them harboring disruptions of all 3 target sites. Our sequence analysis confirms that multiple gRNAs targeting the same locus withing a few hundred base pairs from each other indeed produce a loss of DNA fragments in between corresponding cut sites. Altogether, this data demonstrate that CRISPR-TRiM is a robust method that efficiently knocks out *RNase Z*.

### Loss of cardiac RNase Z causes larval lethality and cardiomyopathy

To tailor CRISPR-TRiM for heart analysis, Cas9 was expressed under control of the robust cardiac-specific 4xtinC^Δ4^ enhancer [22]. We confirmed expression of Cas9 protein by Western blotting (S1A Fig). To generate heart specific *RNase Z* KO we crossed *4xtinC*^*Δ4*^*-Cas9* flies with those expressing 3xgRNA^Z^ and studied their offspring (*tinC-Cas9*>*RNZ*^*KO*^). Control animals (*tinC-Cas9*>*gZ*^*+*^) were produced in the cross between *4xtinC*^*Δ4*^*-Cas9* and *U6-3xgRNA*^*Z*^;*gZ*^*+*^*-PAM*^*Δ*^ flies. In the absence of cardiac RNase Z, larvae develop slower, reaching the body size and weight of wandering third instar on day 7 AED compared to day 5 AED for control animals (S1B and S1D Fig). Majority of *tinC-Cas9*>*RNZ*^*KO*^ larvae (93%) do not pupariate and remain in prolonged 3^rd^ instar for up to 20-day AED when they eventually die. The 7% escapers die 1–2 days after pupariation. Thus, cardiac RNase Z is required for larval development and viability.

**Fig 2:**
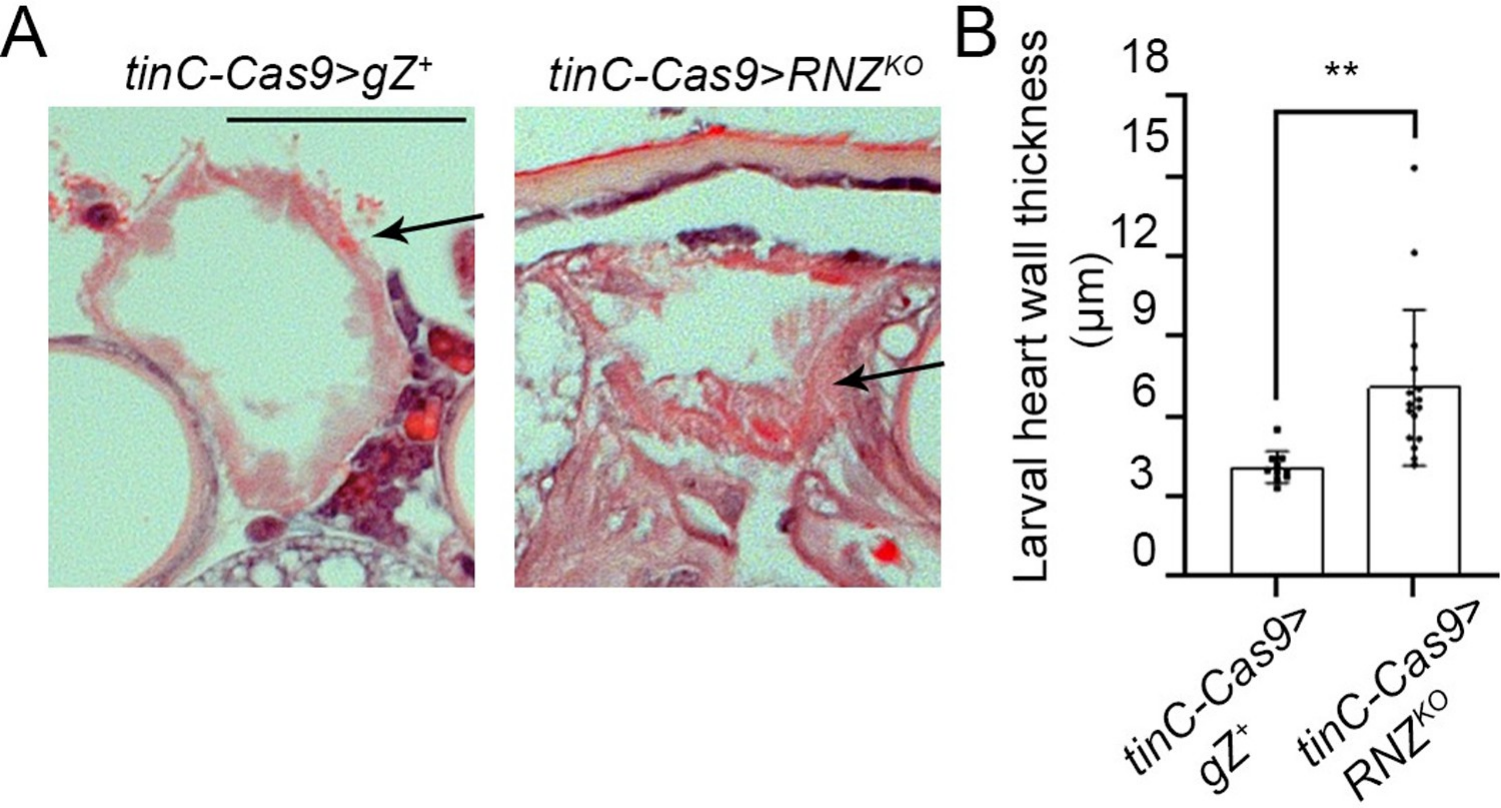
Heart specific RNase Z KO causes heart hypertrophy. (**A**) Histological sections in transverse orientations showing heart wall thicknesses of control *tinC-Cas9*>*gZ*^*+*^ and cardiac-specific mutant larvae *tinC-Cas9*>*RNZ*^*KO*^. Representative images are shown for A6/A7 abdominal segments of the heart of 3^rd^ instar larvae. Arrows point at the heart wall. Scale bar: 50μm. (**B**) Quantification of heart wall thicknesses measured from serial transverse histological sections of larvae *tinC-Cas9*>*gZ*^*+*^ (*n* = 10) and *tinC-Cas9*>*RNZ*^*KO*^ (*n* = 16). **P<0.025 (one-way ANOVA followed by Dunnett’s multiple comparison’s test). Error bars indicate the mean ± s.e.m.

We proceeded to analyze the cell-autonomous requirement for RNase Z in the heart by investigating cardiac morphology and function in *tinC-Cas9*>*RNZ*^*KO*^ larvae. We used a previously described histological approach to measure the heart wall thickness of the 3^rd^ instar. For this, the whole animal was placed in paraffin and sliced 5 microns thick in transverse orientation through the A6/A7 segment where the widest part of the larval heart is located [22]. To account for the variable thickness of the heart wall around its circumference, dorsal, ventral, and lateral measurements were collected from three consecutive tissue slices and used to calculate the average heart wall thickness for each animal. We established that the *tinC-Cas9*>*RNZ*^*KO*^ larvae had a profound heart wall hypertrophy; their heart wall thickness was increased by 100% compared to hearts of control *tinC-Cas9*>*gZ*^*+*^ larvae at the same developmental stage (Fig 2A and 2B).

**Fig 3:**
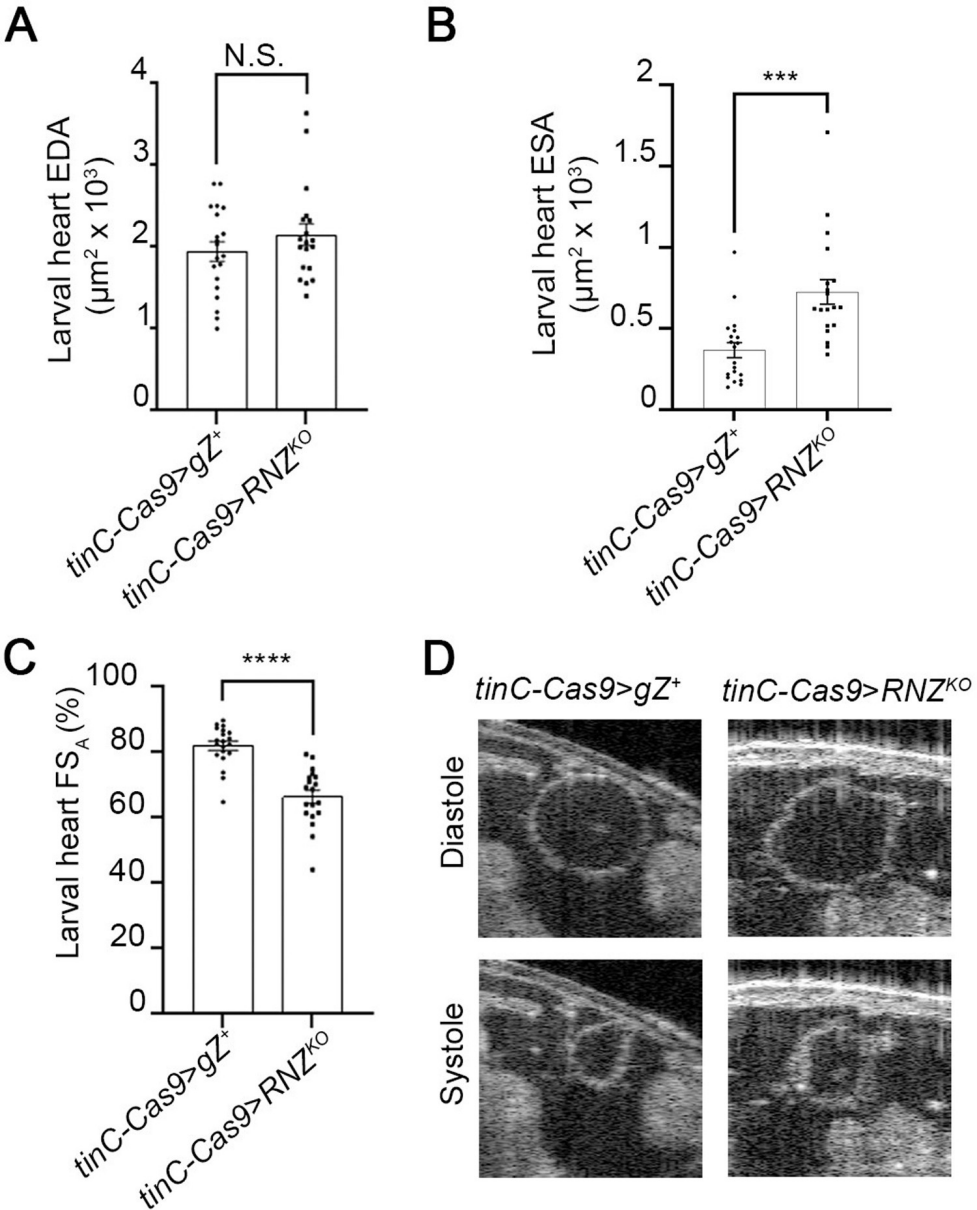
Heart specific RNase Z KO impairs heart contractility. (**A,B,C**) Functional analysis of 3^rd^ instar larval hearts. End Diastolic Area (EDA) in (**A**), End Systolic Area (ESA) in (**B**) and Fractional Shortening (FS_A_) in (**C**) are shown for control *tinC-Cas9*>*gZ*^*+*^ and cardiac-specific mutant *tinC-Cas9*>*RNZ*^*KO*^ larvae (for each genotype, *n* = 20). N.S., no statistical significance; ***P<0.01, ****P<0.001 (one-way ANOVA followed by Dunnett’s multiple comparison’s test). Error bars indicate the mean ± s.e.m. (**D**) OCM images of diastole and systole in A6/A7 heart segment of *tinC-Cas9*>*gZ*^*+*^ and *tinC-Cas9*>*RNZ*^*KO*^ larvae.

Using a non-invasive Optical Coherence Microscopy (OCM) technology we assessed the effect of cardiac specific RNZ^KO^ on heart function (Fig 3 and S1 and S2 Movies). Recorded videos of beating larval hearts in transverse orientation in the A6/A7 segment were used to analyze heart lumen area at the end diastole (EDA) phase, when the heart is the most expanded, and at the end systole (ESA) phase, when the heart is the most contracted. Based on the difference between these two values we calculated the Fractional Shortening (FS_A_), which is a measure of heart contractility [31]. We found that at diastole, *tinC-Cas9>RNZ*^*KO*^ hearts showed some trend of enlargement, which however was not statistically significant when compared to control larvae *tinC-Cas9>gZ*^*+*^ (Fig 3A and 3D). Importantly, these same hearts were hugely dilated by ∼98% at systole (Fig 3B and 3D), which signifies a notable reduction in heart contractility as evidenced by the reduced FS_A_ values by 19% (Fig 3C). Overall, these results demonstrate that cardiomyocyte-specific loss of RNase Z leads to larval heart hypertrophy accompanied by systolic dysfunction and reduced heart contractility, in a cell autonomous manner.

### Missense alleles of *RNase Z* cause cardiomyopathy in a cell autonomous manner

Our analysis of cardiac specific RNase Z loss-of-function animals provides valuable insights into the requirement for this enzyme in *Drosophila* heart. However, given that all known cases of *ELAC2*-associated CM are caused by missense rather than knockout alleles [32], we decided to repeat tissue specific analysis but this time using the hypomorphic mutations of *RNase Z*. Two of such mutations, F154L and T520I were identified in patients with established family history of CM [8, 10]. In our previous study we used these alleles to generate a fly model that recapitulates main symptoms of CM patients and, thus, proves a cause-and-effect connection between *ELAC2* variants and CM [22]. To assess the cell autonomous action of RNase Z variants, we developed a new approach of spatially restricted mutagenesis that coupled tissue-specific CRISPR-Cas9 mediated KO with transgenic rescue. A similar protocol was recently proposed by Chilian and co-authors [33]. An important distinguishing feature of our approach is that instead of Gal4/UAS overexpression as a rescue system, we supply disease-associated protein variants in amounts defined by the activity of the natural *RNase Z* promoter. First, we created two constructs carrying Cas9-resistant alleles of *RNase Z* homologous to human CM-linked mutations–*gZ*^*F155L*^*-PAM*^*Δ*^ and *gZ*^*T494I*^*-PAM*^*Δ*^ (Fig 1B). Using Western blot analysis, we confirmed stable expression of the new RNase Z variants in respective transgenic lines (Fig 1C). Next via fly crossing, we produced two lines each homozygous for two transgenes– *U6-3xgRNA*^*Z*^;*gZ*^*F155L*^*-PAM*^*Δ*^ and *U6-3xgRNA*^*Z*^;*gZ*^*T494I*^*-PAM*^*Δ*^. Finally, to generate mosaic flies that carry a mutant variant of RNase Z only in cardiomyocytes, flies of those two lines were mated with the *4xtinC*^*Δ4*^*-Cas9* flies. Except for cardiomyocytes, all cells in the offspring of these crosses, *tinC-Cas9*>*gZ*^*F155L*^ and *tinC-Cas9*>*gZ*^*T494I*^, have two forms of the RNase Z protein–the wild-type encoded by the endogenous gene and the mutant form encoded by transgenes *gZ*^*F155L*^*-PAM*^*Δ*^ or *gZ*^*T494I*^*-PAM*^*Δ*^, respectively. In the heart however, the endogenous *RNase Z* is knocked out via CRISPR-TRiM, leaving cardiomyocytes in possession of only mutant protein versions–gZ^F155L^ or gZ^T494I^.

**Fig 4:**
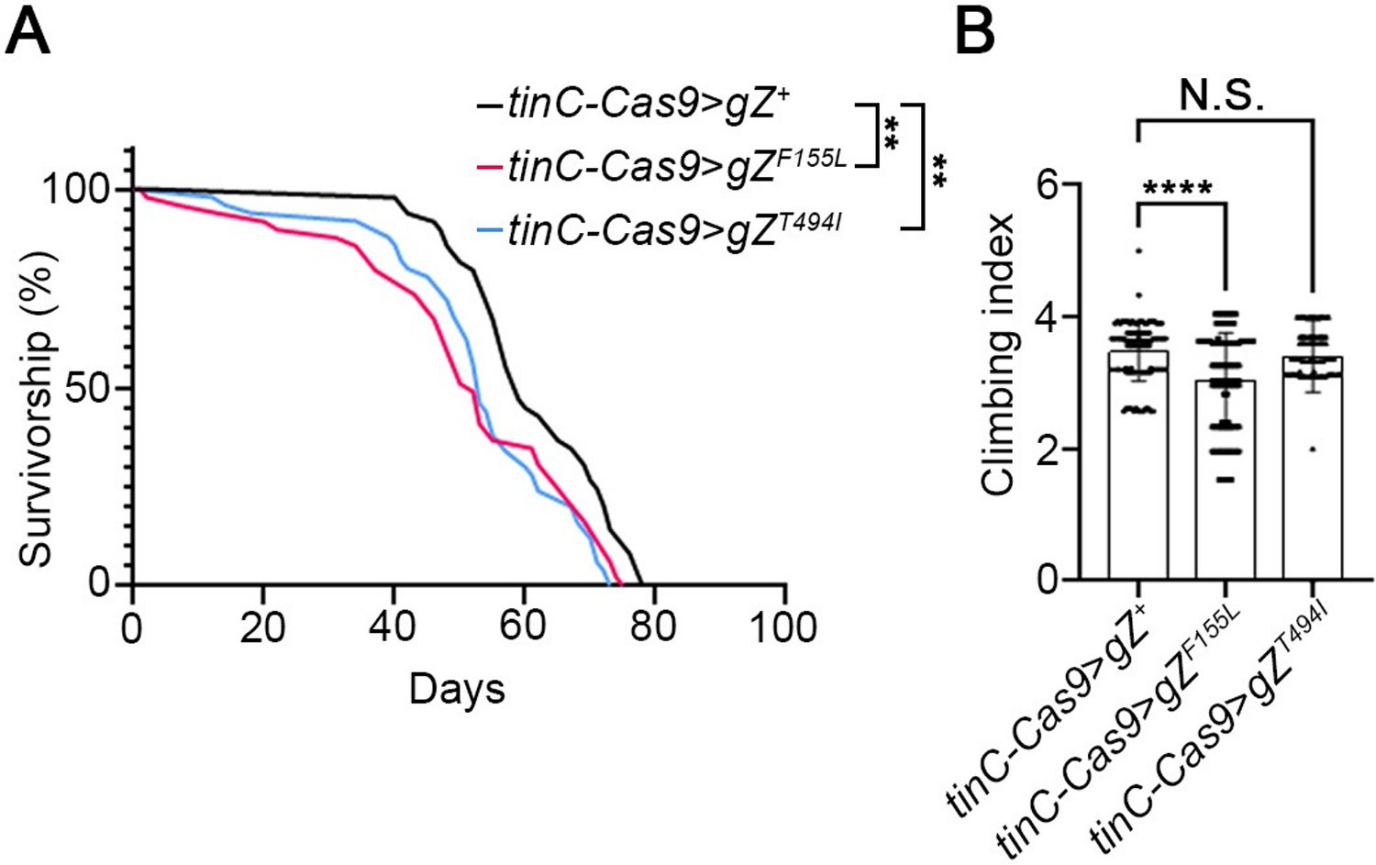
Heart specific RNase Z variants show a mild decrease in longevity and locomotor response. (**A**) Adult longevity was assessed by daily counting live flies over time. Shown are survival rates for control *tinC-Cas9*>*gZ*^*+*^ and cardiac-specific mutant–*tinC-Cas9*>*gZ*^*F155L*^, *tinC-Cas9*>*gZ*^*T494I*^ adult flies (for each genotype, *n* = 50). **P<0.025 (Mantel-Cox test). (**B**) Negative geotaxis expressed as a climbing index is shown for control *tinC-Cas9*>*gZ*^*+*^ and mutant–*tinC-Cas9*>*gZ*^*F155L*^, *tinC-Cas9*>*gZ*^*T494I*^ flies (for each genotype, *n* > 70). N.S., no statistical significance; ****P<0.001 (one-way ANOVA followed by Dunnett’s multiple comparison’s test). Error bars indicate the mean ± s.e.m.

Previously we showed that systemic expression of CM-linked RNase Z variants leads to reduction of fly longevity and locomotor ability [22]. Here we sought to find out if lifespan and fitness were still lessened by RNase Z variants confined to cardiomyocytes. It turned out that flies with heart specific expression of *gZ*^*F155L*^ and *gZ*^*T494I*^ alleles exhibit a mild but statistically significant reduction in longevity. The median lifespan is 46 days for *tinC-Cas9*>*gZ*^*F155L*^ flies and 53 days for *tinC-Cas9*>*gZ*^*T494I*^ flies compared to 54 days for *tinC-Cas9*>gZ^+^ control flies (Fig 4A). For fitness, we measured the locomotor activity of 9-day-old mutant flies using the negative geotaxis assay. Our data show that only *tinC-Cas9*>*gZ*^*F155L*^ flies exhibit reduction in climbing index (by 13%) while *tinC-Cas9*>*gZ*^*T494I*^ flies have climbing index comparable to the control (Fig 4B).

**Fig 5:**
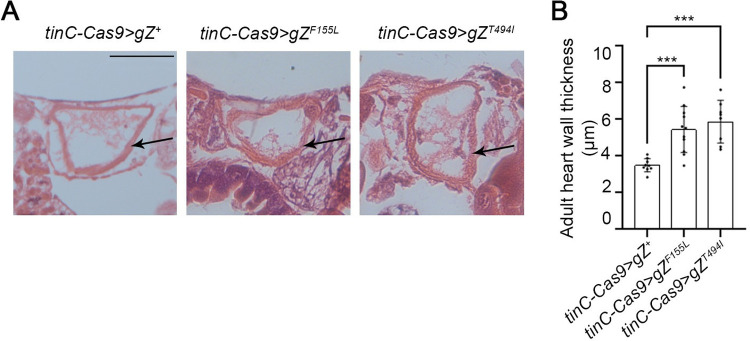
Heart specific RNase Z variants cause heart hypertrophy. (**A**) Histological sections in transverse orientations showing heart wall thicknesses of control *tinC-Cas9*>*gZ*^*+*^ and cardiac-specific mutant flies *tinC-Cas9*>*gZ*^*F155L*^ and *tinC-Cas9*>*gZ*^*T494I*^. Representative images are shown for A1/A2 abdominal segments of the heart of young adults (6–9 days after eclosion). Arrows point at the heart wall. Scale bar: 50μm. (**B**) Quantification of heart wall thicknesses measured from serial transverse histological sections of adult flies *tinC-Cas9*>*gZ*^*+*^ (*n* = 9), *tinC-Cas9*>*gZ*^*F155L*^ (*n* = 11) and *tinC-Cas9*>*gZ*^*T494I*^ (*n* = 8). ***P<0.01 (one-way ANOVA followed by Dunnett’s multiple comparison’s test). Error bars indicate the mean ± s.e.m.

We also studied cell-autonomous effects of cardiac RNase Z variants on heart morphology and contractility. Using histological approach, we measured the cardiac wall thickness in the A1/A2 segment where adult heart is the widest. Just like in larvae, we collected multiple measurements around the circumference of adult heart to account for the variable thickness of the wall. We found that *tinC-Cas9*>gZ^*F155L*^ and *tinC-Cas9*>gZ^*T494I*^ flies had an increase in heart wall thickness by 60% and 68% respectively, compared to the *tinC-Cas9*>gZ^+^ controls (Fig 5A and 5B).

**Fig 6:**
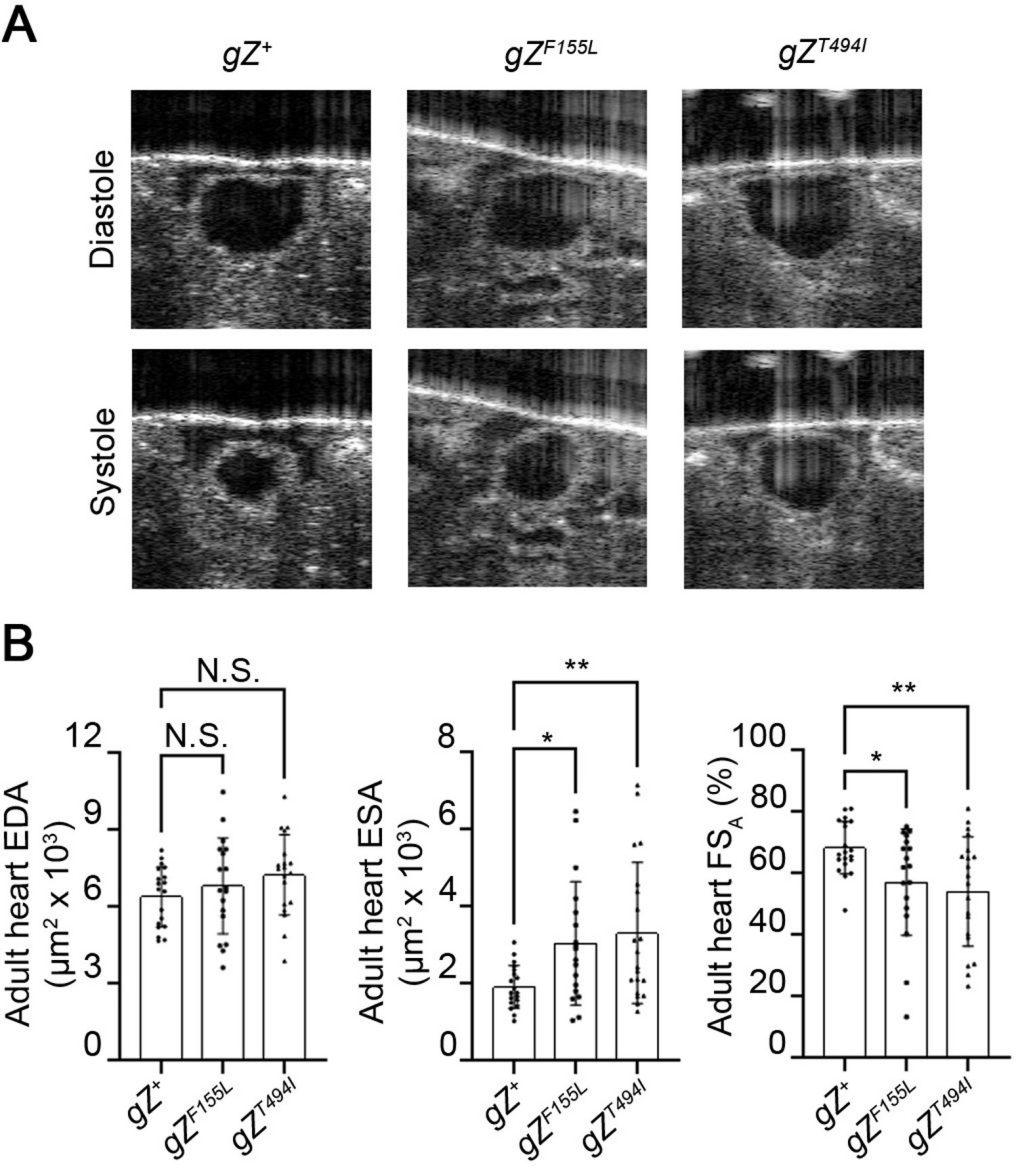
Heart specific RNase Z variants impair heart contractility. (**A**) OCM images of diastole and systole in A1/A2 heart segment of control *gZ*^*+*^ (*tinC-Cas9*>*gZ*^*+*^) and cardiac-specific mutant–*gZ*^*F155L*^ (*tinC-Cas9*>*gZ*^*F155L*^), *gZ*^*T494I*^ (*tinC-Cas9*>*gZ*^*T494I*^) adult flies. Flies of all genotypes were studied 6–9 days after eclosion. (**B**) Functional analysis of adult fly hearts. EDA, ESA and FS_A_ are shown for *gZ*^*+*^, *gZ*^*F155L*^ and *gZ*^*T494I*^ adult flies (for each genotype, *n* = 20). N.S., no statistical significance; *P<0.05, **P<0.025 (one-way ANOVA followed by Dunnett’s multiple comparison’s test). Error bars indicate the mean ± s.e.m.

Next, we studied adult heart function, using the OCM method, which allowed us to visualize heart contractions in a non-invasive manner. We measured EDA and ESA in the A1/A2 segment of adult hearts in transverse orientation. Heart specific expression of RNase Z variants caused dilation of heart lumen at systole by 60% for *tinC-Cas9*>gZ^*F155L*^ and 73% for *tinC-Cas9*>*gZ*^*T494I*^ flies, which led to a reduction in heart contractility by 16% in *tinC-Cas9*>*gZ*^*F155L*^ and 20% in *tinC-Cas9*>*gZ*^*T494I*^ flies (Fig 6A and 6B and S3–S5 Movies). These results show that heart hypertrophy and reduction in contractility are caused by RNase Z variants in a cell autonomous manner.

## Discussion

### CRISPR/Cas9-mediated knockout of *RNase Z* is specific and efficient

Our goal was to eliminate *RNase Z* expression in a controlled manner in order to understand its role in heart development and function. As a model, *Drosophila* offers a variety of genetic tools for gene disruption. To eliminate *RNase Z* gene expression, we decided to use the versatile CRISPR/Cas9 gene editing system [23]. Several considerations led us to CRISPR/Cas9 over other commonly used techniques such as the FLP/FRT recombination system [34], RNA interference (RNAi) [35], ZFN- and TALEN-based mutagenesis [36, 37]. The FLP/FRT system generates a mosaic tissue with twin spots of homozygous mutant and wild-type cells which does not suit our objective of introducing *RNase Z* KO in every cell of the heart. RNAi approach is simple and straightforward in practice; however, it does not produce a complete knockout, but rather a knockdown with variable efficiency depending on the target gene. Moreover, because of possible partial base pairing of siRNAs, this approach is prone to the off-target effect [38]. ZFN- and TALEN-mediated gene knock out techniques are similar to CRISPR/Cas9 as they also rely on double-strand DNA breaks coupled with error-prone repair. The specificity of these nuclease-based techniques depends on their DNA-binding domains, which could be customized to recognize a particular target sequence. Thus, simultaneous targeting of multiple sites would require the engineering of several forms of ZFN and/or TALEN, which significantly complicates the design. CRISPR/Cas9, on the other hand, is guided to a specific location in the genome by a small gRNA. Thus, creating multiple DSBs within a specific locus does not require multiple Cas9 nucleases but only several gRNAs, which is much easier to design.

While the CRISPR/Cas9 approach appeared to perfectly fit our goal, there were several concerns related to its application. First, as a mutagen, CRISPR/Cas9 relies on the cellular DNA repair mechanisms that do not produce uniform modification at the DSB site in every cell. The error-prone NHEJ-mediated repair introduces small indels and nucleotide substitutions at the target site. In some cells these modifications may lead to amorphic alleles via frame-shift or nonsense mutations, and yet in others–to hypomorphic alleles via missense mutations. As a result, the tissue, in which CRISPR/Cas9 is activated, ends up being mosaic and composed of cells carrying different mutations, which could complicate the analysis. To avoid this issue and to achieve a genuine KO in each cardiac cell, we employed not one but three gRNAs. They were designed to target a 600-bp fragment within the 5’ end of *RNase Z* coding region, where genetic modifications would have the highest functional impact [24, 25]. To confirm the efficiency of our design, we combined multiplexed 3xgRNA^Z^ with ubiquitously expressed Cas9. All larvae carrying two transgenes– *U6-3xgRNA*^*Z*^ and *Act5C-Cas9* –died, which indicated that expression of the essential *RNase Z* gene was successfully disrupted.

The second concern of CRISPR/Cas9 application is the off-target effects. Even with significantly improved scoring algorithms and gRNA selection tools, a small possibility of nonspecific genome modification still remains [39]. In this study, we used a two-pronged approach to confirm the specificity of *RNase Z* knock out. First, by simple analysis of mutant phenotypes we found a striking consistency in the lethal phases of *Act-Cas9*>*RNZ*^*KO*^ animals and those carrying *RNaseZ* null *Z*^*24*^ allele. Both groups display early larval lethality, as most of them die soon after first larval molt, 3 days AED (this study and [26]). This observation gave us some confidence that the phenotype of *Act-Cas9*>*RNZ*^*KO*^ animals is caused by specific knock out of *RNase Z* gene expression via CRISPR/Cas9. Still, the ultimate proof that a particular phenotype results from inactivation of a specific gene is the rescue experiment with the same gene product *in vivo*. Several techniques employed in the past made use of CRISPR/Cas9-resistant rescue constructs encoding either a putative ortholog with diverged nucleotide sequence or the endogenous gene in which target regions are modified with frequent silent nucleotide mismatches. However, in case of CRISPR/Cas9 the success of these strategies is questionable because of the notorious tolerance of the system towards guide-target mismatches. In this study, we created the CRISPR/Cas9 resistant rescue construct by introducing single-nucleotide substitutions in three PAM sites [40]. These modifications are critical as they allow the *gZ*^*+*^*-PAM*^*Δ*^ transgene to escape the Cas9-mediated cleavage. When we combined this construct with *Act5C-Cas9* and *U6-3xgRNA*^*Z*^, it rescued animals to adulthood, confirming that the early larval lethality phenotype was due to the specific knockout of *RNase Z* expression.

Assuming that targeting a gene with multiple gRNAs might increase the chances of mutagenesis, many studies used multiplexed gRNAs for achieving gene knockout [41, 42]. Curiously, the primary method for evaluating the outcome of the multi-gRNA activity was phenotypic analysis via scoring offspring lethality [43, 44]. Only few studies have looked at the underlying genomic modifications to confirm a knockout. And even these studies evaluated the multiplex approach indirectly–either by following the expression of the GFP reporter [23], or by sequencing amplicons around the gRNA target sites [25]. In the latter case, the absence of two gRNA targets was viewed as the loss of the intervening sequences. Here in our study, to evaluate the KO of *RNase Z*, we looked at both lethality and the underlying molecular modifications. The expectation was that by employing three gRNAs targeting the 600-bp region within the 5’ half of *RNase Z*, we might produce a loss of large DNA fragments rather than small indels at individual target sites. To determine the status of CRISPR-Cas9 induced DNA damage, we PCR-amplified the whole 600-bp region with primers located ∼350 bp 5’ and 3’ to the flanking gRNA target sites (Fig 1A). By sequencing 100 randomly selected gDNA fragments we found the spectrum of deficiencies, ranging from ∼200 to ∼1200 bp. Importantly, 90% of deletions were losses of 600 bp or larger with the complete removal of the entire target region. A closer look at these deletions showed that most of them encompassed all three target sites. Our analysis illustrates the advantage of applying multiplexed gRNA with the 100% efficiency (this study) compared to single gRNA with the reported 23% efficiency of mutagenesis [33]. Taken together, the sequencing data and the lethal phenotype of the *tub-Cas9*>*RNZ*^*KO*^ larvae, support the expectation that multiple gRNAs targeting the same locus within a few hundred base pairs from each other produce large deficiencies resulting in knockout mutations.

### Cardiac specific knockout of RNase Z

In general, loss of RNase Z activity in endoreplicating tissues results in cell growth deficiency. Previous studies showed that either knockdown of RNase Z in salivary glands or a complete knockout of its expression within somatic clones in larval fat body caused a dramatic reduction in cell size [26, 45]. Here, however, we found that post-mitotic endocycling cardiac tube deprived of RNase Z undergoes severe hypertrophy. The apparent inconsistency with prior data is caused by the timing of RNase Z inactivation and the availability of maternal supply of the enzyme. In the fat body, RNase Z expression was disrupted during early embryogenesis, just a few hours after egg deposition. At that time, the developing embryo relies solely on the maternal gene products. As this source is depleted, there is no more RNase Z owing to the interrupted zygotic expression, which leads to halted fat body cell growth. In our current research involving cardiomyocyte-specific RNase Z, disruption occurs only during late embryogenesis at 10–11 hrs AED under the control of the *4xtinC*^*Δ4*^ enhancer we employed. By this developmental time point, zygotic *RNase Z* expression has been uninterrupted for multiple hours; adequate levels of enzyme could have been produced to process the tRNA molecules and thus aid in cell growth.

One of the first notable observations that we made in animals devoid of cardiac RNase Z is their lethality; none of them survived to adulthood. This raises the question of whether the heart itself is essential for fly viability or not. On one hand, it has been shown that to some extent *Drosophila* can tolerate the loss of the heart. The heart-specific knockdown of a ribosomal protein RpL13 led to the absence of the heart tube structures. Interestingly, some animals with “no heart” phenotype were able to progress into the adult stages [46]. On the other hand, cardiomyocyte-specific disruption of genes involved in actin dynamics and histone modifications, resulted in 100% pre-adult mortality [47]. Adding our own data, we suggest that there is some evidence that fly heart is an essential organ for life.

Importantly, when we looked at the heart of larvae with myocardium-specific loss of RNase Z, we discovered gross structural and functional abnormalities. The heart wall was twice as thick as that of the control animals, and the myocardial contraction was inefficient as the heart tube was still dilated by 98% in systole. These results are consistent with our previous finding that linked RNase Z to cardiac hypertrophy [22]. In an attempt to understand why null alleles of this gene cause heart overgrowth, we looked at the molecular function of the protein. RNase Z is an endonucleolytic enzyme that processes nuclear and mitochondrial precursor tRNA (pre-tRNA) 3’ ends [48]. It has been established that RNase Z knockout abrogates mitochondrial transcript processing, increases reactive oxygen species (ROS) and induces a switch to aerobic glycolysis compensating for cellular ATP [49]. We suggest that heart hypertrophy and impairment that we observed in *tinC-Cas9*>*RNZ*^*KO*^ larvae is due to mitochondrial distress. Indeed, cases of cardiomyopathies that stem from defects in mitochondrial function have been well documented through clinical studies [50]. Moreover, mitochondrial cardiomyopathy has been reproduced and studied in several model organisms. In zebrafish, a knockout of mito-tRNA modifying enzyme MTO1 causes multiple mitochondria flaws, which result in hypertrophy of cardiomyocytes and myocardial fiber disarray [51]. SOD2 is an essential antioxidative enzyme scavenging ROS and protecting mitochondria; mice with reduced SOD2 activity develop heart hypertrophy and display an increase in left ventricular volume in systole [52]. Mitochondria activity depends on mitochondrial gene expression, which is affected when ELAC2 and/or organelle-specific RNase P endonucleases are inactivated. Mice lacking ELAC2 or mitochondrial RNase P in the heart have a short life span and die with profound cardiomyopathy [53, 54]. Given the indispensable role of *Drosophila* RNase Z in mitochondrial transcript processing [49] and in view of multiple cases of mitochondrial cardiomyopathy being documented, it is reasonable to suggest that heart hypertrophy and disfunction that we observed in RNase Z KO animals is due to impaired mitochondrial activity. Yet, the specific molecular pathway that would connect mitochondria to heart failure remains unknown.

A different mechanism underlying heart pathology in *tinC-Cas9*>*RNZ*^*KO*^ larvae could be drawn from studies suggesting a link between cardiac hypertrophy and non-coding RNA (ncRNA) [55, 56]. In particular, there is a new and fast-growing class of ncRNA called tRFs–tRNA-derived fragments. Those are represented by three types: tRF-5, tRF-3 and tRF-1 [57]. The first two originate from the 5’ and 3’ ends of mature tRNA, respectively. The tRF-1 group originates from the pre-tRNA 3’-trailer released by RNase Z endonucleolytic cleavage. Importantly, a robust increase in tRF levels was observed in hearts with induced hypertrophy in rats [58]. Furthermore, mutant *Drosophila* flies with defective tRNA biogenesis display alterations in tRF populations [59]. In mice, heart-specific knockout of RNase Z impairs tRNA processing resulting in imbalanced levels of different types of tRFs; mutant mice develop a severe form of cardiomyopathy and die early [54]. More studies are needed to identify mechanistic insights into the association between regulatory tRFs and myocardium hypertrophy.

### Cardiac specific RNase Z variants promote CM

Multiple studies from different labs have highlighted the importance of *Drosophila* for the identification and analysis of genes relevant to human cardiac pathophysiology [60, 61]. Given a high degree of conservation in genes that control heart development and function between *Drosophila* and mammals, researchers performed cardiac specific RNAi silencing to identify genes whose inactivation would impair cardiomyocyte contractility [47, 62–66]. We used a different approach, as we started with a particular human gene, *ELAC2*, for which missense mutations were correlated with the occurrence of an aggressive form of infantile CM [8, 10, 11, 32]. We validated a causal connection between *ELAC2* mutant alleles and CM using *Drosophila* homolog *RNase Z* [22]. Our strategy was based on replacing rather than silencing the endogenous fly gene with the mutant *RNase Z* variants analogous to CM-causing alleles in human patients. With this model, we showed that mutant flies did recapitulate major symptoms, including thicker heart wall, dilated heart lumen, and decreased cardiac contractility [22]. Besides CM, those flies exhibited other traits such as reduced fitness, shortened lifespan, and neurodegeneration. Pleiotropy as a phenomenon associated with *RNase Z* is not surprising as the gene is expressed ubiquitously and activity of the RNase Z protein is indispensable in many cell types [26, 45, 67]. However, dissecting complex phenotypes with the goal to identify the key processes leading to heart failure could be a daunting task, as malfunction of one tissue can influence the severity of damage in other tissues.

In this study, we investigated the cell autonomous role of RNase Z in the *Drosophila* heart. By employing a novel approach of CRISPR-TRiM mediated KO coupled with transgenic rescue, we generated a mosaic fly wherein only cardiomyocytes carry pathogenic alleles in place of the wild-type RNase Z. Our study is the first attempt to characterize the cell autonomous response to CM-associated mutations in vivo. Importantly, we found that cardiac-restricted expression of the F155L and T494I mutant alleles was sufficient to compromise heart function (Figs 5 and 6). Because of systolic dilation, fractional shortening was reduced by 16% in *tinC-Cas9*>*gZ*^*F155L*^ and 20% in *tinC-Cas9*>*gZ*^*T494I*^ flies (Fig 6B). Given that similar reduction of heart contractility– 17% and 23%–was observed in flies with systemic expression of *gZ*^*F155L*^ and *gZ*^*T494I*^ [22], it appears that this damage arises solely from cardiomyocyte intrinsic cell-autonomous processes. Interestingly, a similar conclusion cannot be made about myocardium hypertrophy, as cardiac-specific mutants produced a 60% to 68% increase in heart wall thickness (Fig 5) compared to 111–123% observed in systemic mutants *gZ*^*F155L*^ and *gZ*^*T494I*^ [22]. This indicates that in addition to cell autonomous there are nonautonomous processes that contribute to the hypertrophic response. One of those could be deposition of Pericardin (PRC), an insect collagen IV-like protein. PRC is a prominent ECM component; synthesized and secreted by the fat body cells, it is deposited exclusively around *Drosophila* cardiac tube. A recent study described an inter-organ communication circuitry that modulates cardiac ECM; pericardial cells activated by elevated ROS levels release cytokine Upd3, which triggers fat body to express and produce larger amounts of PRC [68]. Indeed, previously we observed that hypertrophy in *gZ*^*F155L*^ and *gZ*^*T494I*^ systemic mutants is accompanied by higher levels of PRC around the heart [22]. Knowing that *Drosophila* cells deprived of RNase Z accumulate ROS [49], it is reasonable to suggest that pericardial cells expressing mutant alleles would communicate the cytokine signal to the fat body and initiate fibrosis. However, in the mosaic flies described in this study, expression of *gZ*^*F155L*^ and *gZ*^*T494I*^ alleles is restricted to the cardiomyocytes, which leaves pericardial cell unaffected and hypertrophic hearts without excessive PRC deposition.

Previous studies showed that flies with compromised heart activity display reduced lifespan [69]. Not surprisingly, when we tested longevity of mutants with systemic expression of *gZ*^*F155L*^ and *gZ*^*T494I*^ alleles, we found dramatic reduction in median lifespan down to 6–11 days [22]. In the current study the same mutations, being restricted to cardiomyocytes in *tinC-Cas9*>*gZ*^*F155L*^ and *tinC-Cas9*>*gZ*^*T494I*^ flies, produced a much milder effect. Their median lifespan was almost normal: 46–53 days compared to 54 days for the *tinC-Cas9*>*gZ*^*+*^ control flies (Fig 4A). A similar observation was made with respect to the locomotor activity, as cardiac-specific mutants displayed only 0% to 13% reduction in climbing index (Fig 4B) compared to 78–88% reduction observed in flies with systemic mutations [22]. Put together, these results suggest that the impact of cardiac-specific expression of two hypomorphic *RNase Z* alleles on longevity and fitness of adult flies is very mild if any at all. Apparently, a strong reduction in both traits previously observed in the systemic mutants is a response to impaired function of a different tissue or even a cumulative response to multiple functions of several tissues/organs being impaired. Overall, our data corroborates the pleiotropic effect of RNase Z and highlights the advantage of models with tissue-specific expression of its mutant forms in unravelling cell autonomous and nonautonomous mechanisms underlying the pathogenesis of congenital CM.

## Materials and methods

### Fly stocks

Flies were maintained at 25°C on standard cornmeal-molasses-agar medium. The following flies from the Drosophila Stock Center (Bloomington, IN, USA) were used in this study: *Act-Cas9* (#54590), and *tub-Cas9* (#81930). The dRNaseZ knockout flies (Z^24^) and transgenic lines carrying gZ^+^-PAM^+^ (also referred to as genZ^+^-V5) were described previously [26]. Table 3 provides genotypes and detailed description of key fly stocks generated in this study.

**Table 3:** Experimental models.

Complete genotype	Abbreviation	Description
{Act5C-Cas9.P}/+;{U6-3xgRNA^z^}/+	*Act-Cas9>RNZ* ^*KO*^	Ubiquitous knockout of endogenous RNase Z
{Act5C-Cas9.P}/+;{U6-3xgRNA^z^}/+;gZ^+^-PAM^Δ^	*Act-Cas9>gZ* ^*+*^	Ubiquitous knockout of RNase Z rescued with Cas9 resistant wild type RNase Z transgene
{4xtinC^Δ4^-Cas9/{U6-3xgRNA^z^}	*tinC-Cas9>RNZ* ^*KO*^	Cardiac knockout of endogenous RNase Z
{4xtinC^Δ4^-Cas9/{U6-3xgRNA^z^};{gZ^+^-PAM^Δ^}/+	*tinC-Cas9>gZ* ^*+*^	Cardiac knockout of RNase Z rescued with Cas9 resistant wild type RNase Z transgene
{4xtinC^Δ4^- Cas9/{U6-3xgRNA^z^};{gZ^F155L^-PAM^Δ^}/+	*tinC-Cas9>gZ* ^*F155L*^	Cardiac knockout of RNase Z rescued with Cas9 resistant F155L variant of RNase Z transgene
{4xtinC^Δ4^- Cas9/{U6-3xgRNA^z^};{gZ^T494I^-PAM^Δ^}/+	*tinC-Cas9>gZ* ^*T494I*^	Cardiac knockout of RNase Z rescued with Cas9 resistant T494I variant of RNase Z transgene

### DNA cloning and generation of transgenic fly strains

To express Cas9 under control of a cardiac specific promoter, we used the 4xtinC^Δ4^ promoter that was generated and described previously [22]. Briefly, this promoter features four tandem repeats of the cardiac enhancer tinCΔ4, from the tinman gene [70]. A pair of primers 5’-aaaggaaccaattcagtcgaCGAATTGGGTACTCTAGAC-3’ and 5’-caagaaagctgggtctagatAAAAGCTGGAGCTCCAC-3’ were used to PCR amplify the promoter from the 4xtinC^Δ4^-pBSc plasmid serving as a template. The 4xtinC^Δ4^ fragment was cloned into the SalI and EcorV sites of the Gateway pENTR3C vector (ThermoFisher, A10464) using the NEBuilder HiFi DNA Assembly master mix (New England BioLabs), and then transferred into the pDest-APIC-Cas9 expression vector (Addgene, 121657) by an LR reaction using Gateway™ LR Clonase™ II Enzyme mix (ThermoFisher, 11791020). Transgenic line carrying 4xtinC^Δ4^-Cas9 on the second chromosome at the 57F5 cytological region was generated using phiC31-mediated site-specific integration (Genetivison). Transgene expression was confirmed by Western blot with anti-Cas9 antibodies (S1A Fig).

For targeted *RNaseZ* disruption, we used the flyCRISPR optimal target finder online tool ((http://targetfinder.flycrispr.neuro.brown.edu/). Three gRNAs with the highest score and without any off-targets were selected and cloned under the ubiquitous promoter employing strategy described in [23]. Briefly, we used a gRNA PCR template vector pMGC (gift from Dr. Han, Cornell University, Ithaca, NY) to generate PCR products containing (F+E) gRNA scaffold and tRNA^Gly^ as well as *RNaseZ* specific target sequences. The two primer pairs that we used are: 5’-TTCCCGGCCGATGCA**TGTTCGCAGACACACGTTGC**GTTTAAGAGCTATGCTG GAAACAG-3’ with 5’-**GGCTGATTTTACTAAATACA**TGCACCAGCCGGGAATC-3’ and 5’-**TGT ATTTAGTAAAATCAGCC**GTTTAAGAGCTATGCTGGAAACAG-3’ with 5’-TTCCAGCATAGCT CTTAAAC**CGAAACGTCGCATTGACTGC**TGCACCAGCCGGGAATC-3’ (letters in bold indicate Target Sequences 1–3). Using HiFi DNA Assembly kit, we introduced the PCR products into SapI sites of pAC-U63-tgRNA-Rev expression vector (gift from Dr. Han, Cornell University, Ithaca, NY). To establish a transgenic line, we used phiC31-mediated site-specific integration to insert the transgene into the AttP site on the second chromosome at 57F5 (GenetiVison).

To generate an RNase Z variant that is resistant to Cas9, we sequentially introduced a single-nucleotide substitution into each of the three PAM sites (Fig 1B) within the genRNZ-V5 expression construct, which we created previuosly [26]. The genRNZ-V5 is the pCa4B2G transformation vector that carries a 6.6-kb fragment of genomic DNA containing the complete coding region for RNase Z tagged with the V5-epitope along with the 3-kb upstream and 1-kb downstream sequences. Single nucleotide substitutions were introduced with the Q5 site-directed mutagenesis Kit (New England Biolabs) using the following pairs of primers: 5’-ACACGTTGCC**T**GTGCTGACGA-3’ with 5’-GTCTGCGAACAGCTGTTGC-3’ for PAM1, 5’-TCTACAGAACCTTGCGAACG-3’ with 5’-TCGGCGA**T**CTGGCTGATTTTAC-3’ for PAM2 and 5’-CGTTTCGT**C**GTGCTAAAGAATC-3’ with 5’-TCGCATTGACTGCAGCATAG-3’ for PAM3 (letters in bold indicate introduced nucleotide changes), yielding gZ^+^-PAM^Δ^-V5 construct. Next, we introduced either one of CM-linked mutations into gZ^+^-PAM^Δ^-V5, using Q5 site-directed mutagenesis kit (New England Biolabs). We used following pairs of primers to introduce 594T>C, and 1669C>T mutations: 5’-AATGCGACGT**C**TCGTCGTGCT-3’ with 5’-GACTGCAGC ATAGAGCCGAG-3’ for gZ^F155L^ and 5’-TAAAATCAGCCAGATCGC-3’ with 5’-CTGACGAAATAC GA**T**TGTATTTAG-3’ for Z^T494I^ (letters in bold indicate introduced nucleotide changes), yielding gZ^F155L^-PAM^Δ^-V5 and gZ^T494I^-PAM^Δ^-V5 constructs. *E*. *coli* transformants were studied and successful cloning was confirmed by restriction digestion and sequencing. To establish transgenic lines, we used phiC31-mediated site-specific integration to insert the transgenes into the AttP site on the third chromosome at 68A4 (Genetivison). Transgene expression was confirmed by Western blot with anti-V5 antibodies (Fig 1C).

### Western blot analysis

For adult fly analysis, protein extracts were prepared by homogenizing five whole animals in Laemmli sample buffer. All samples were boiled for 3 min. Samples were separated on an SDS-polyacrylamide gel and subjected to Western blotting. Blots were incubated overnight at 4°C with primary antibodies diluted in PBST supplemented with 5% dry milk powder (w/v). Antibodies used were anti-V5 (Invitrogen, 1:10,000), anti-α-tubulin (Sigma, 1:5000), anti-Cas9 (Invitrogen, 1:10,000). Visualization of blots was done using KwikQuant Digital Western Blot Detection System (Kindle Biosciences). Band densities were quantified using the KwikQuant imaging software.

### Genomic DNA analysis

For the analysis of variability in CRISPR/Cas9 mediated modifications, genomic DNA (gDNA) was extracted from 100 tub-Cas9>RNZ^KO^ larvae (NucleoSpin Tissue kit, Macherey‑Nagel). The 1377-bp fragment containing the entire target region along with the ∼350-bp upstream and downstream sequences was amplified on the gDNA template with the following primers: 5'-AACGCACGTGGGCATTAGAG-3' and 5'-TAAGGCGTGAAGTGCACCAC-3' (Fig 1A), using Q5 Polymerase (New England Biolabs). PCR fragments were purified (QIAquick PCR Purification kit, Qiagen) and cloned into the pGEM-T Easy Vector (Promega). 100 clones were randomly selected and sequenced unidirectionally with M13 primer that anneals to the vector (Azenta Lifesciences) to study genomic modifications.

### Longevity assay

Longevity was measured as previously described [22]. Briefly, adult males and females of indicated genotypes were collected on the day of eclosion and placed into same-sex cohorts of ten flies per vial. The number of survivors in each vial was scored daily. The flies were placed in fresh vials three times per week (Monday, Wednesday, and Friday) during the entire test. Statistical analysis was performed using Mantel-Cox test.

### Negative geotaxis assay

Flies’ locomotor activity was tested using the geotaxis assay as previously described [71, 72]. Flies were collected on the day of eclosion under brief CO_2_ anesthesia (1–2 min), sorted in groups of 10 (5 males and 5 females) and allowed to recover at least 18 h at 25°C prior to the assay. Next day, flies were transferred into vials marked with lines forming four equally spaced quadrants. Each vial was gently tapped to bring flies to the bottom of the vial and initiate the negative geotaxis response. Flies were allowed to climb up for four seconds before a photograph is taken to record position of each fly in the vial. Each fly was assigned a rating based on the quadrant it reached; flies that did not climb and stayed at the bottom were scored as zero. Weighted average of three consecutive trials was calculated for each vial and represented a climbing index for that vial.

### Histological analysis

To ensure all animals are studied at the same developmental point, we synchronized them by behavioral and morphological criteria as described elsewhere [45]. Fly heart wall thickness was measured as previously described [22]. Briefly, 3^rd^ instar larvae of mixed sexes were fixed in FAAG solution (80% EtOH, 5% Acetic acid, 4% Formaldehyde, 1% Gluteraldehyde) for 24hrs at 4°C.). Adult flies (6-9d old females) were fixed in Carnoy’s fixative overnight at 4°C. After fixation, both larval and adult samples were dehydrated through increasing gradient of EtOH solutions, washed with xylenes and then placed into hot paraffin. Solidified paraffin blocks were sliced in transverse orientation at 5μm thickness; sliced sections were placed on slides for subsequent Hematoxylin and Eosine staining.

Sections were rehydrated and stained with Hematoxylin and Eosin (Sigma). All histological samples were analyzed under the Zeiss Axio Imager M1 microscope, brightfield images were captured with AxioCam MRc camera. Wall thickness was measured (ImageJ) and calculated as an average of three measurements from each section, in three consecutive sections of the heart.

### Optical coherence microscopy

Fly heart contraction was measured as previously described [22, 73–75]. Briefly, a superluminescent diode (cBLMD-T-850-HP, Superlum, Ireland) with a central wavelength of ∼850 nm and a bandwidth of ∼170 nm was used as the light source for the OCT system. The axial and transverse resolutions provided by the system were ∼3.3 μm and ∼2.8 μm in tissue, respectively. The backscattered light from the reference and sample arms was detected using a 2048-pixel spectrometer (CS800-840/180-80-OC2K-U3, Wasatch Photonics, USA), operated at 20k A-scans/s. Sensitivity of the system was determined to be ∼ 95 dB. M-mode images which repeated scanning at the same xz plane were acquired at a frame rate of ∼126 Hz. Flies were mounted on a glass slide by a double-sided tape (larvae) or glue (adult files) with the dorsal side exposed for imaging.

Larval hearts were studied in A6/A7 segments. We measured end-diastolic areas (EDAs) and end-systolic areas (ESAs) of 30 consecutive best visible heartbeats per larva, using ImageJ (National Institutes of Health, USA). Fractional shortening (FSA) was calculated as (EDA-ESA)/EDA×100%.

Adult hearts were studied at the transition of A1-to-A2 segment (∼50 μm from the posterior end of the thorax), where the heart chamber is widest and best visible. Usually, adult heart contractions display some variability over time. To account for that, we collected EDA and ESA measurements from the heart beats spaced at three-second intervals throughout the 30 seconds of time-lapse OCM images, which gave us 10 impartially selected heart beats from each recording.

### Statistical analysis

All graphs and statistical analyses were performed in Microsoft Excel and GraphPad Prism9. Statistical data are presented as mean ± standard error of the mean (s.e.m). P values for all the comparisons was determined by one-way ANOVA followed by Dunnett’s multiple comparison’s test unless specified. The mean difference was considered statistically significant at the 95% confidence level. Results were considered as not significant (ns) when P>0.05, significant when 0.01<P< 0.05 (*), very significant when 0.001<P<0.025 (**) and extremely significant when P<0.001 (***) or P <0.0001 Figures were assembled with Adobe Photoshop (Adobe Systems, San Jose, CA).

## Supporting information

S1 Fig Generation and characterization of Cardiac-specific RNaseZ knockout. A. Expression of tinC-Cas9. Western blot analysis of proteins extracted from larval hearts of tinC-Cas9 and Act-Cas9 transgenic animals. **B, C, D. Loss of cardiac RNaseZ delays larval development. B**. Representative images of WT control (U6-3xgRNA^Z^) larvae at 5d AED and tinC-Cas9>RNZ^KO^ larvae at 5d and 7d AED **C.** Body length of U6-3xgRNA^Z^ at 5d AED, and tinC-Cas9>RNZ^KO^ larvae at 5d and 7d AED (n = 10). **D.** Body mass of U6-3xgRNA^Z^ at 5d AED, and tinC-Cas9>RNZ^KO^ larvae at 5d and 7d AED (n = 3 repeats with 10 larvae measured each repeat).
(TIF)

S1 Movie M-mode video of tinC-Cas9>gZ^+^ beating heart in 3rd instar. Video is captured around A7 segment.
(MP4)

S2 Movie M-mode video of tinC-Cas9>RNZ^KO^ beating heart in 3rd instar. Video is captured around A7 segment.
(MP4)

S3 Movie M-mode video of 7-day old tinC-Cas9>gZ^+^ beating adult heart. Video is captured around A1 segment.
(MP4)

S4 Movie M-mode video of 7-day old tinC-Cas9>gZ^F155L^ beating adult heart. Video is captured around A1 segment.
(MP4)

S5 Movie M-mode video of 7-day old tinC-Cas9>gZ^T494I^ beating adult heart. Video is captured around A1 segment.
(MP4)

S1 Raw images Raw images for Western blots presented in Figs 1C and S1A. (PDF)
