# Supplementary figures and images for: Cardiac RNase Z edited via CRISPR-Cas9 drives heart hypertrophy in Drosophila
Source: PLoS One. 2023 May 25;18(5):e0286214. doi: 10.1371/journal.pone.0286214 (PMC10212119; doi:10.1371/journal.pone.0286214)

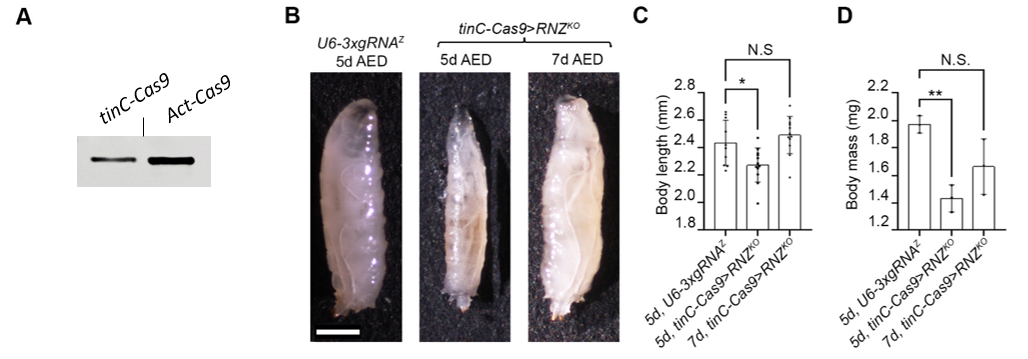

Supplement: S1 Fig — Western blot analysis of proteins extracted from larval hearts of tinC-Cas9 and Act-Cas9 transgenic animals. B, C, D. Loss of cardiac RNaseZ delays larval development. B. Representative images of WT control (U6-3xgRNAZ) larvae at 5d AED and tinC-Cas9>RNZKO larvae at 5d and 7d AED C. Body length of U6-3xgRNAZ at 5d AED, and tinC-Cas9>RNZKO larvae at 5d and 7d AED (n = 10). D. Body mass of U6-3xgRNAZ at 5d AED, and tinC-Cas9>RNZKO larvae at 5d and 7d AED (n = 3 repeats with 10 larvae measured each repeat). (TIF) [file pone.0286214.s001.tif]
