# Supplementary material for: Cardiac RNase Z edited via CRISPR-Cas9 drives heart hypertrophy in Drosophila
Source: PLoS One. 2023 May 25;18(5):e0286214. doi: 10.1371/journal.pone.0286214 (PMC10212119; doi:10.1371/journal.pone.0286214)

S1 Raw images of Western blots for figures 1C and S1A

Raw images of blots pertaining to Figure 1C “**Western blot analysis of RNase Z proteins whose expression is driven by indicated transgenes**”. The white^{1118} stock flies are used as a negative control; α-Tubulin is a loading control. RNase Z is detected with the anti-V5 antibody. The lane marked with an “X” is not part of the current manuscript, and hence was not included in the final figure.

Anti-V5

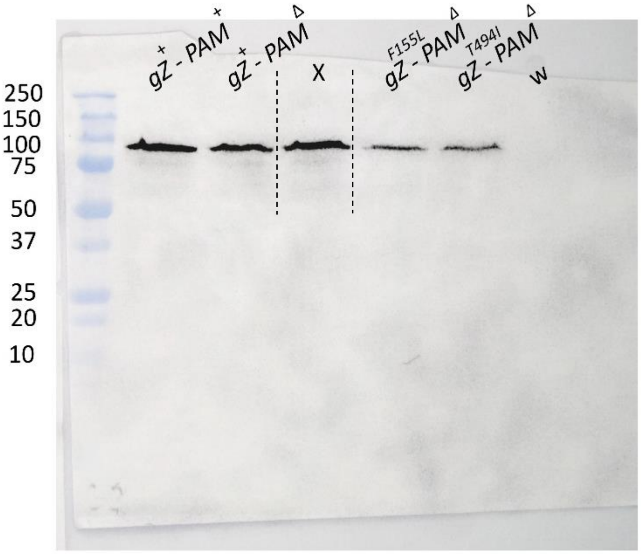

Anti-Tubulin

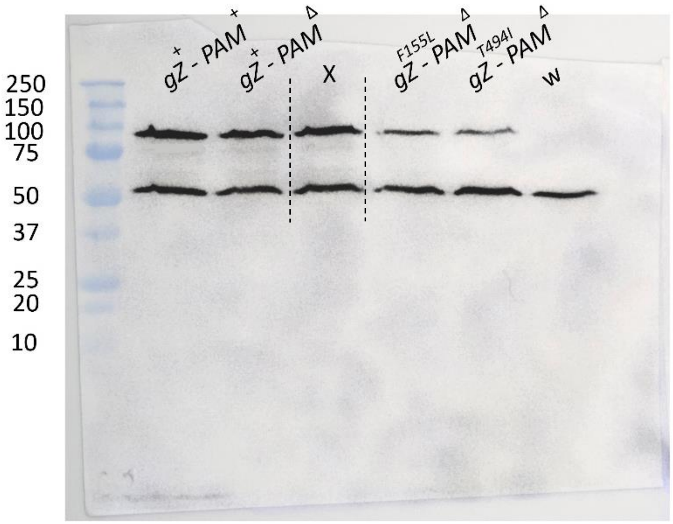

Raw image of blot pertaining to Figure S1A “**Expression of tinC-Cas9**”. Act-Cas9 larvae were used as a positive control. The Cas9 proteins from larval hearts were detected using anti-Cas9 antibodies. The lanes marked with an “X” are not part of the current manuscript, and hence was not included in the final figure.

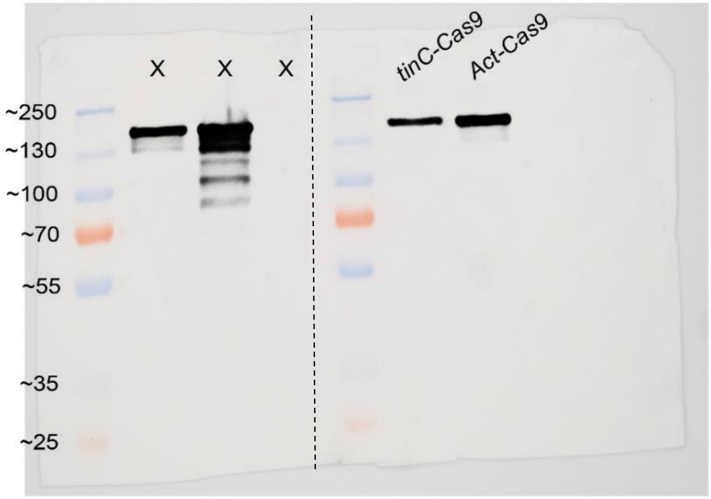

Supplement: S1 Raw images — (PDF) [file pone.0286214.s007.pdf]
